# Supplementary figures and images for: Health impact assessment and cost‒benefit analysis: Exploring complementarities of methods to assess the impacts of regulations on food consumption
Source: PLoS One. 2025 Jul 1;20(7):e0326946. doi: 10.1371/journal.pone.0326946 (PMC12212541; doi:10.1371/journal.pone.0326946)

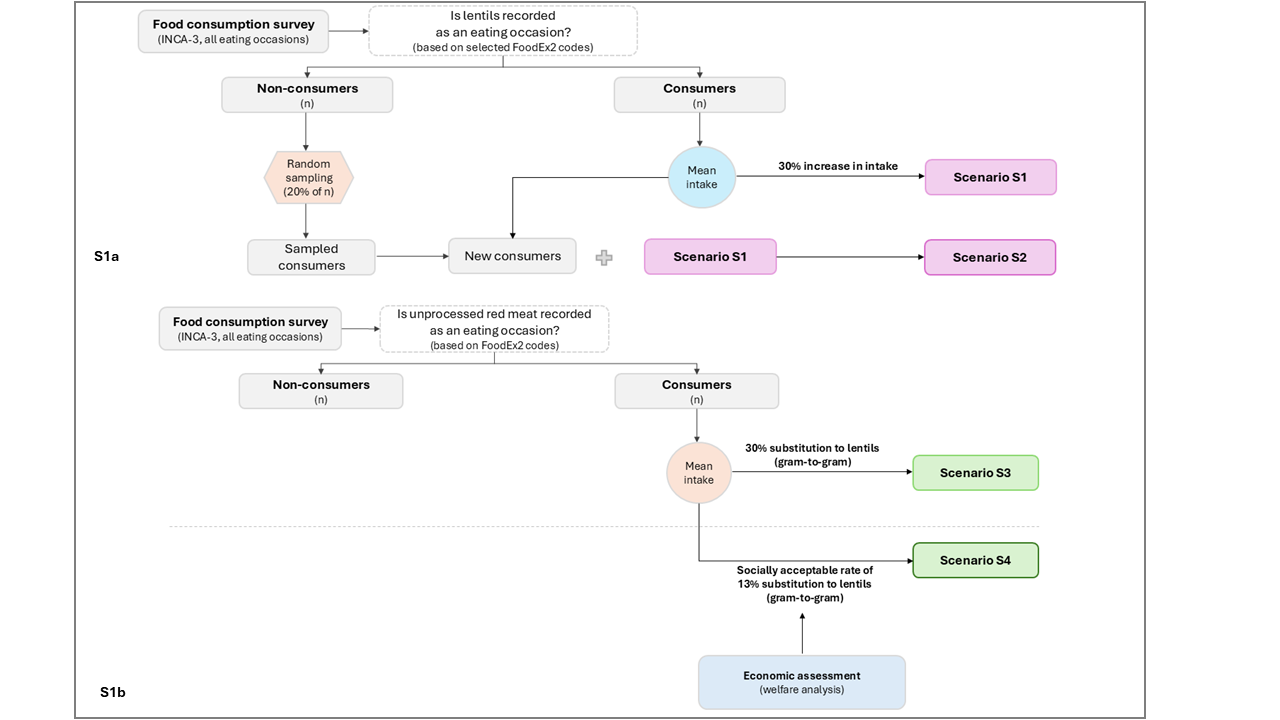

Supplement: S1 Fig — (TIF) [file pone.0326946.s001.tif]
