# Supplementary material for: Health impact assessment and cost‒benefit analysis: Exploring complementarities of methods to assess the impacts of regulations on food consumption
Source: PLoS One. 2025 Jul 1;20(7):e0326946. doi: 10.1371/journal.pone.0326946 (PMC12212541; doi:10.1371/journal.pone.0326946)
Supplement: S1 File — (DOCX) [file pone.0326946.s002.docx]

**Supplementary material_1**

**Table S1. FoodEx2 codes used to filter consumption data.**

| **FoodEx2^a^** | |
| --- | --- |
| A01BD | Canned or jarred lentils |
| A0DCE | Lentils (dry) and similar |
| A013Q | Lentils (dry) |
| A01QV | Bovine fresh meat |
| A01QX | Cow, ox or bull fresh meat |
| A01QY | Calf fresh meat |
| A049S | Bovine, minced meat |
| ^a^ Food classification standardisation system (EFSA, 2015) | |

**Table S2. Selected health outcomes included in the RBA applications.**

| **Food** | **Component** | **Health effect** | **Health impact metric** | **Application cases^a^** |
| --- | --- | --- | --- | --- |
| Lentil | Whole food | Reduced risk of IHD | DALY | Example 1 and 2 |
| Red meat | Whole food | Increase risk of CRC | DALY | Example 2 |
| Red meat | Whole food | Increase risk of T2DM | DALY | Example 2 |
| Lentil | Cadmium | Increase risk of CKD | DALY | Example 1 and 2^b^ |
| Lentil and red meat | Iron | Reduced risk anaemia (microcytic) | DRVs comparison^c^ | Example 1 and 2 |
| Lentil and red meat | Selenium | Reduced risk of deficiency | DRVs comparison | Example 1 and 2 |
| Lentil and red meat | Zinc | Reduced risk of deficiency | DRVs comparison | Example 1 and 2 |
| Red meat | Vitamin B12 | Reduced risk of anaemia (macrocytic) | DRVs comparison | Example 2 |
| IHD, ischaemic heart disease; CKD, chronic kidney disease; CRC, colorectal cancer; T2DM, diabetes mellitus type II; DRV, dietary reference values.  ^a^ Example 1 refers to the consumption scenarios S1-S2, example 2 refers to S3-S4. ^b^ Results not presented in the paper. Mean daily cadmium exposure in the general French adult population was 0.043 (95% uncertainty intervals: 0.009-0.113) µg/kg bw/day. Following a bottom-up approach, this exposure yielded 0 DALYs. ^c^ As stablished by EFSA; Results only presented for S3 since S1-S2 do not account for food substitutions and results of S3 could be extrapolated for S4. | | | | |

**Table S3. Data sources used in the RBA for computing DALYs.**

| **Variable type** | **Data** | **Reference** |
| --- | --- | --- |
| Food consumption and micronutrient composition | French Individual and National Food Consumption Survey (INCA3, 2014-2015) | (Dubuisson et al., 2019)^a^ |
| Chemical concentration in foods | French Total Diet Study 2  (TDS-France, 2006-2007) | (ANSES, 2011; Redondo et al., 2023)^b^ |
| Conversion factor | Dietary to urinary Cadmium (Cd) | (Amzal et al., 2009) |
|  | Reduction in glomerular filtration rate due to Cd exposure | (Ginsberg, 2012) |
| Dose-response | Ischemic heart disease (IHD) | (Afshin et al., 2014) |
|  | Colorectal cancer (CRC) | (WCRF, 2018) |
|  | Diabetes mellitus type 2 (T2DM) | (Pan et al., 2011) |
| Dietary Reference Values for nutrients (DRVs) | Average requirement (AR) or adequate intake (AI) for micronutrients | (EFSA, 2019) |
| DALY envelope and incidence cases | Global Burden of Disease 2019, estimates for France | (IHME, 2020) |
| Population demographics | Demographic balance 2019 | (Insee, 2020) |
| ^a^ Publicly available data (<https://www.data.gouv.fr/fr/datasets/donnees-de-consommations-et-habitudes-alimentaires-de-letude-inca-3/>). ^b^ Concentration data set extracted from the work of Redondo et al. (2023) | | |

**References**

Afshin, A., Micha, R., Khatibzadeh, S., & Mozaffarian, D. (2014). Consumption of nuts and legumes and risk of incident ischemic heart disease, stroke, and diabetes: A systematic review and meta-analysis. In *American Journal of Clinical Nutrition* (Vol. 100, Issue 1, pp. 278–288). American Society for Nutrition. https://doi.org/10.3945/ajcn.113.076901

Amzal, B. , Julin, B. , Vahter, M. , Wolk, A. , Johanson, G. , & Åkesson2, A. ,. (2009). Population Toxicokinetic Modeling of Cadmium for Health Risk Assessment. *Environmental Health Perspectives*, *117*(8).

ANSES. (2011). *Second French Total Diet Study (TDS 2) Report 1 Inorganic contaminants, minerals, persistent organic pollutants, mycotoxins and phytoestrogens ANSES Opinion*.

Dubuisson, C., Dufour, A., Carrillo, S., Drouillet-Pinard, P., Havard, S., & Volatier, J. L. (2019). The Third French Individual and National Food Consumption (INCA3) Survey 2014-2015: Method, design and participation rate in the framework of a European harmonization process. In *Public Health Nutrition* (Vol. 22, Issue 4, pp. 584–600). Cambridge University Press. https://doi.org/10.1017/S1368980018002896

EFSA. (2015). The food classification and description system FoodEx 2 (revision 2). *EFSA Supporting Publications*, *12*(5). https://doi.org/10.2903/sp.efsa.2015.EN-804

EFSA. (2019). *DRV Finder*. https://multimedia.efsa.europa.eu/drvs/index.htm

Ginsberg, G. L. (2012). Cadmium risk assessment in relation to background risk of chronic kidney disease. *Journal of Toxicology and Environmental Health - Part A: Current Issues*, *75*(7), 374–390. https://doi.org/10.1080/15287394.2012.670895

IHME. (2020). *GBD Results*. Seattle, WA: IHME, University of Washington. https://vizhub.healthdata.org/gbd-results/

Insee. (2020). *Demographic balance sheet 2019*. https://www.insee.fr/en/statistiques/2382597?sommaire=2382613

Pan, A., Sun, Q., Bernstein, A. M., Schulze, M. B., Manson, J. A. E., Willett, W. C., & Hu, F. B. (2011). Red meat consumption and risk of type 2 diabetes: 3 Cohorts of US adults and an updated meta-analysis. *American Journal of Clinical Nutrition*, *94*(4), 1088–1096. https://doi.org/10.3945/ajcn.111.018978

Redondo, H. G., Guillier, L., Bemrah, N., Jakobsen, L. S., Thomsen, S. T., & Pires, S. M. (2023). Harmonized approach to estimate the burden of disease of dietary exposure to four chemical contaminants - A French study. *Science of the Total Environment*, *894*. https://doi.org/10.1016/j.scitotenv.2023.164804

WCRF. (2018). *Diet, nutrition, physical activity and colorectal cancer*. https://www.wcrf.org/wp-content/uploads/2021/02/Colorectal-cancer-report.pdf
