# Supplementary material for: Health impact assessment and cost‒benefit analysis: Exploring complementarities of methods to assess the impacts of regulations on food consumption
Source: PLoS One. 2025 Jul 1;20(7):e0326946. doi: 10.1371/journal.pone.0326946 (PMC12212541; doi:10.1371/journal.pone.0326946)
Supplement: S3 File — (PDF) [file pone.0326946.s004.pdf]

```
In[1]:= p1T = 10.4
p2S = 2.7
qT = 60144810
qS = 19665319

BT = 1.11 * qT / p1T
GG = 0.02 * qS / p1T
AT = qT + BT * p1T - GG * p2S
BSS = 1.23 * qS / p2S
ASS = qS + BSS * p2S - GG * p1T

Valx = Simplify[{at, bet, g, as, bes} /.
    Solve[{AT == (at bes - as g)/(bes bet - g^2), BT == bes/(bes bet - g^2), GG == g/(bes bet - g^2),
       ASS == (as bet - at g)/(bes bet - g^2), BSS == bet/(bes bet - g^2)}, {at, bet, g, as, bes}]];
at = Valx[[1, 1]]
bet = Valx[[1, 2]]
g = Valx[[1, 3]]
as = Valx[[1, 4]]
bes = Valx[[1, 5]]

deH = 0.19
delR = 0.12
H = deH * p2S
R = delR * p1T
pT = 10.4;
pS = 2.7;

MEATCONSUMERSONLY
qBB = 1018757395
BTmm = 1.11 * qBB / p1T
zzGE = BTmm * GG / BT
AMz = qBB + BTmm * p1T - zzGE * p2S
BLL = BTmm * BSS / BT
ALLz = 0 + BLL * p2S - zzGE * p1T

Mxx = Simplify[{amm, bmm, gmm, ale, ble} /.
    Solve[{AMz == (amm ble - ale gmm)/(ble bmm - gmm^2), BTmm == ble/(ble bmm - gmm^2), zzGE == gmm/(ble bmm - gmm^2),
       ALLz == (ale bmm - amm gmm)/(ble bmm - gmm^2), BLL == bmm/(ble bmm - gmm^2)}, {amm, bmm, gmm, ale, ble}]];
amm = Mxx[[1, 1]]
bmm = Mxx[[1, 2]]
gmm = Mxx[[1, 3]]
ale = Mxx[[1, 4]]
ble = Mxx[[1, 5]]

WelfareViandeLentille
eff2 = as * xeqS - (pS - s) * xeqS - (bes * xeqS^2)/2 + at * xeqT -
   (pT + t) * xeqT - (bet xeqT^2)/2 - g xeqT * xeqS + H * xeqS - R * xeqT;
ee1i = at - bet * xT - g * xS;
```

```
ee2i = as - bes * xS - g * xT;
uxi = Simplify[{xT, xS} /. Solve[{ee1i == pT + t, ee2i == pS - s}, {xT, xS}]];
Weli = Simplify[eff2 /. {xeqT -> uxi[[1, 1]], xeqS -> uxi[[1, 2]]}];
WelfareforMeatOnly
eff33 = ale * xeqSUU - (pS - s) * xeqSUU - (ble * xeqSUU^2)/2 + amm * xeqTXX -
    (pT + t) * xeqTXX - (bmm xeqTXX^2)/2 - gmm xeqTXX * xeqSUU + H * xeqSUU - R * xeqTXX;
ee1Y = amm - bmm * xTt - gmm * xSs;
ee2Y = ale - ble * xSs - gmm * xTt;
uxiVV = Simplify[{xTt, xSs} /. Solve[{ee1Y == pT + t, ee2Y == pS - s}, {xTt, xSs}]];
Weli33 = Simplify[eff33 /. {xeqTXX -> uxiVV[[1, 1]], xeqSUU -> uxiVV[[1, 2]]}];
Budget
tax1 = +t * uxi[[1, 1]] + t * uxiVV[[1, 1]] - s * uxi[[1, 2]] - s * uxiVV[[1, 2]]
Welfare
welpr = Simplify[Weli + Weli33 + tax1]

ECOWELFARE
OptiTAXSubvention
uSpr = {t, s} /. Solve[{D[welpr, t] == 0, D[welpr, s] == 0}, {t, s}]
welfaretaxe
welFIFI = N[welpr /. {t → R, s → H }]
Welfaresansrien
welFx = N[welpr /. {t → 0, s → 0}]
Variation Quantite
vian = N[uxi[[1, 1]] + uxiVV[[1, 1]] /. {t → R, s → H }];
lent = N[ uxi[[1, 2]] + uxiVV[[1, 2]] /. {t → R, s → H }];
viande
variviande = vian - qT - qBB
rela = variviande / (qT + qBB)
lentille
varilent = lent - qS
rela2 = varilent / (qS)
Variationwelfare
delvvvv = N[welFIFI - welFx]
ral33 = delvvvv / welFx

WelfareDaly
xeqTyy = qT * 0.7;
xeqSyy = qS + qT * 0.3;
eff2yy = as * xeqSyy - (pS) * xeqSyy - (bes * xeqSyy^2)/2 + at * xeqTyy -
    (pT) * xeqTyy - (bet xeqTyy^2)/2 - g xeqTyy * xeqSyy + H * xeqSyy - R * xeqTyy;
xeqTXXxx = qBB * (0.7);
xeqSUUxx = qBB * (0.3);
eff33yy = ale * xeqSUUxx - (pS) * xeqSUUxx - (ble * xeqSUUxx^2)/2 + amm * xeqTXXxx -
    (pT) * xeqTXXxx - (bmm xeqTXXxx^2)/2 - gmm xeqTXXxx * xeqSUUxx + H * xeqSUUxx - R * xeqTXXxx;
WWW = eff2yy + eff33yy
Variation Quantite
viande
ccvv = - (qT * 0.3 + qBB * (0.3))
```

```
lentille
rrr = qT * 0.3 + qBB * (0.3)
rela = rrr / qS
Variationwelfare
delvvvv22 = Expand[WWW - welFx]
rela = delvvvv22 / welFx
```

Out[1]= 10.4

Out[2]= 2.7

Out[3]= 60144810

Out[4]= 19665319

Out[5]= 6.4193×10^{6}

Out[6]= 37817.9

Out[7]= 1.26803×10^{8}

Out[8]= 8.95865×10^{6}

Out[9]= 4.34604×10^{7}

Out[11]= 19.7825

Out[12]= 1.55784×10^{-7}

Out[13]= 6.57625×10^{-10}

Out[14]= 4.93473

Out[15]= 1.11627×10^{-7}

Out[16]= 0.19

Out[17]= 0.12

Out[18]= 0.513

Out[19]= 1.248

Out[22]= MEATCONSUMERSONLY

Out[23]= 1018757395

Out[24]= 1.08733×10^{8}

Out[25]= 640575.

Out[26]= 2.14785×10^{9}

Out[27]= 1.51745×10^{8}

Out[28]= 4.0305×10^{8}

Out[30]= 19.7696

Out[31]= 9.19709×10^{-9}

Out[32]= 3.88245×10^{-11}

Out[33]= 2.73955

Out[34]= 6.59016×10^{-9}

Out[35]= WelfareViandeLentille

General::spell1 :
Possible spelling error: new symbol name "xeqT" is similar to existing symbol "xeqS". Plus…

Out[41]= WelfareforMeatOnly

Out[47]= Budget

Out[48]= $(1.01876\times10^{9} - 640575.\ s - 1.08733\times10^{8}\ t)\ t + (6.01448\times10^{7} - 37817.9\ s - 6.4193\times10^{6}\ t)\ t - s\ (1.96653\times10^{7} + 8.95865\times10^{6}\ s + 37817.9\ t) - s\ (-8.52992\times10^{-9} + 1.51745\times10^{8}\ s + 640575.\ t)$

Out[49]= Welfare

Out[50]= $-8.03519\times10^{7}\ s^{2} + s\ (8.32877\times10^{7} - 678393.\ t) - 5.7576\times10^{7}\ (-9.40761 + t)\ (6.90557 + t)$

Out[51]= ECOWELFARE

Out[52]= OptiTAXSubvention

Out[53]= {{1.248, 0.513}}

Out[54]= welfaretaxe

Out[55]= 3.85168×10^{9}

Out[56]= Welfaresansrien

Out[57]= 3.74042×10^{9}

General::spell1 :
Possible spelling error: new symbol name "Quantite" is similar to existing symbol "Quantile". Plus…

Out[58]= Quantite Variation

Out[61]= viande

Out[62]= -1.44058×10^{8}

Out[63]= -0.133523

Out[64]= lentille

Out[65]= 8.32877×10^{7}

Out[66]= 4.23526

Out[67]= Variationwelfare

Out[68]= 1.11255×10^{8}

Out[69]= 0.0297441

Out[70]= WelfareDaly

```
General::spell1 :
 Possible spelling error: new symbol name "xeqSyy" is similar to existing symbol "xeqTyy". Plus…
```

Out[77]= 3.53339×10^{9}

Out[78]= Quantite Variation

Out[79]= viande

Out[80]= -3.23671×10^{8}

Out[81]= lentille

Out[82]= 3.23671×10^{8}

Out[83]= 16.459

Out[84]= Variationwelfare

Out[85]= -2.07033×10^{8}

Out[86]= -0.0553501
